# Puf4 is methylated and exhibits a temperature-dependent interactome in *Cryptococcus neoformans*

**DOI:** 10.1128/spectrum.02628-25

**Published:** 2026-02-25

**Authors:** Murat C. Kalem, Sean Duffy, Amanda L. M. Bloom, Shichen Shen, Jan Naseer Kaur, Jun Qu, John C. Panepinto

**Affiliations:** 1 Department of Microbiology and Immunology, Witebsky Center for Microbial Pathogenesis and Immunology, Jacobs School of Medicine and Biomedical Sciences, University at Buffalo 12292 , Buffalo, New York, USA; 2 Department of Pharmaceutical Sciences, University at Buffalo 12292 , Buffalo, New York, USA; 3 New York State Center of Excellence in Bioinformatics and Life Sciences 132184 , Buffalo, New York, USA; University of Florida College of Dentistry, Gainesville, Florida, USA

**Keywords:** post-translational modification, complementation, arginine methylation, phosphorylation, fungal pathogen, *Cryptococcus neoformans*, transcriptome, RNA immunoprecipitation, mass spectrometry

## Abstract

The evolutionarily conserved Pumilio domain-containing RNA binding proteins (RBPs) are involved in many steps of post-transcriptional gene regulation, including RNA stability, polyadenylation, deadenylation, and translation. Most RBPs are post-translationally modified by methylation of arginine/glycine-rich domains (RG-box) though the consequences of these modifications are not well known. We determined the arginine methylation and phosphorylation landscape of the Pumilio domain containing RBP Puf4 from the basidiomycete fungus *Cryptococcus neoformans*. We found that methyl-deficient Puf4 mutants do largely complement critical *PUF4* deletion phenotypes, such as resistances to endoplasmic reticulum stress, antifungals, and thermotolerance, but were unable to complement the melanization defect of the *puf4*△ mutant. Analysis of the Puf4 interactome revealed temperature-dependent and -independent protein-protein interactions, some of which were altered by the methyl-deficient mutations. Overall, we show that the RNA-binding protein Puf4 exhibits extensive post-translational modifications, particularly arginine methylation that impacts function and protein-protein interactions.

**IMPORTANCE:**

Post-transcriptional processes play a crucial role in modulating responses to various stressors, including temperature and antifungals. To ensure adaptation and survival, stressors must be accurately sensed, and responses tightly regulated. Post-translational modifications serve as a crucial regulatory layer that orchestrates nuanced stress responses. We highlight extensive methylation and phosphorylation of the RNA-binding protein Puf4 and explore the regulatory roles of select methylated arginines. We also found that the interactome network of Puf4 varies with temperature. Cryptococcus neoformans, a basidiomycete fungus that is also an important human pathogen, is an ideal system to illustrate the evolution of gene regulatory processes and how these kingdom or clade-specific regulatory modules contribute to the pathogenic potential of fungi.

## INTRODUCTION

RNA binding proteins (RBPs) are key post-transcriptional regulators that fine-tune critical cellular events and are instrumental in every step of the RNA life cycle, from synthesis to translation and decay. Misregulation of RBP activity can be pathological, contributing to cancer and neurodegeneration (1–3). The binding domains within RBPs and the cognate RNA features they recognize are evolutionarily conserved across kingdoms, but the processes they regulate and their consequences may be kingdom specific (4, 5). Pumilio and Fem-3 mRNA binding factor (PUF) domains in RBPs, first identified in the arthropod *Drosophila melanogaster* and the nematode *Caenorhabditis elegans* (6–8), contain highly conserved helical repeats that regulate the localization, decay, and translation of specific mRNAs (9, 10). *D. melanogaster* has one PUF domain-containing RBP, humans have two, the basidiomycete fungus *Cryptococcus neoformans* has four, the ascomycete fungus *Saccharomyces cerevisiae* has six, and the nematode *C. elegans* and the kinetoplastid parasite *Trypanosoma cruzi* each have 10 (9–12). These different numbers of PUF proteins suggest evolutionary diversification in the regulatory roles these proteins play (13, 14).

RBPs are dynamically regulated by post-translational modifications (PTM) (15–17). For example, the highly conserved and abundant arginine-glycine (RG)-rich domains in RBPs can be methylated (18, 19). Arginine methylation is catalyzed by arginine methyltransferases (RMTs), which are similarly diverse across the evolutionary landscape (20, 21). The *S. cerevisiae* genome encodes four RMTs, whereas the human genome encodes nine, contrary to their numbers of PUF proteins (six and two, respectively) (22). The apparent inverse association between the numbers of RMTs and PUF proteins led us to hypothesize that functional diversification of gene regulation is driven by the variety of RBPs in less complex organisms and by arginine methylation (and PTMs in general) of RBPs in more complex organisms. The ability to leverage dynamic and often reversible PTMs may impart agility for post-transcriptional gene regulation (23, 24). Our recent work to characterize RMTs in *C. neoformans* revealed that one of its four PUF proteins, Puf4, interacts with Rmt5, suggesting that its function is controlled through methylation (25). Puf4 regulates mRNAs involved in cell wall biosynthesis and is involved in the splicing of *HXL1*, the major endoplasmic reticulum (ER) stress transcription factor (26, 27). Of note, *puf4*△ mutants are resistant to the ER stress-inducing drug tunicamycin and the antifungal drug caspofungin (26–28).

Although PUF proteins have been extensively studied, we are not aware of any studies on the regulation of PUF protein function by PTMs (9, 29, 30). The human PUF protein Pum1 was shown to be methylated, but the functional importance of this modification was not explored (31). In this study, we set out to assess Puf4 PTMs in *C. neoformans* and their functional consequences. *C. neoformans* is a well-established and genetically tractable fungal model organism that has been used to investigate post-transcriptional processes and to showcase their divergence across fungal clades (32–34).

## MATERIALS AND METHODS

### Yeast strains and molecular cloning

Strains used in this study were derived from *Cryptococcus neoformans* var. *grubii* strain H99, a fully virulent strain (gifted by Peter Williamson, UIC, NIAID) derived from strain H99O (gifted by John Perfect, Duke University). The *puf4*∆ mutant and Puf4-FLAG complement cell lines were previously established (26). Methyl-deficient Puf4 constructs were established using a QuickChange site-directed mutagenesis kit (Agilent). All primers used in this study are listed in Table S1. Cell lines were established using biolistic transformation.

### Phylogenetic analysis

A BLAST search was used to find the orthologs of Rmt5 in fungal species from *Ascomycota*, *Basidiomycota*, *Mucoromycotina*, and *Chytridiomycota* phyla by using the *C. neoformans PUF4* gene (CNAG_02810) as the query. All sequences were aligned using the default algorithm of Geneious alignment followed by a neighbor-joining tree protein alignment on Geneious. Human *PUM1* and *PUM2* were included as outgroups.

### Puf4 immunoprecipitation and LC-MS/MS

*For PTM analysis:* FLAG immunoprecipitation was performed as described previously (26). Puf4-FLAG overexpression strain and wild-type (H99) cells were grown to an optical density at 600 nm (OD_600_) of 1.5–1.8 at 30°C in 2 L YPD broth while shaking at 200 rpm. Cells were collected by centrifugation at 7,000 × *g* for 5 min. Pellets were frozen in liquid N_2_ and stored at −80°C. Frozen cell pellets were ground using liquid N_2_ in a coffee grinder (Krups F203) for 3 min. Cell powder was then further ground using a mortar and pestle for 15–20 min with the frequent addition of liquid N_2_. Cell powder was transferred to 50-mL conical tubes and stored at −80°C or immediately used for immunoprecipitation. Cell powder was dissolved in RIP buffer (25 mM HEPES-KOH [pH 7.9], 0.1 mM EDTA, 0.5 mM EGTA, 2 mM MgCl_2_, 20% glycerol, 0.1% Tween-20, 300 mM KCl, 1× cOmplete protease inhibitor tablet [Roche], 50 U/mL RNaseOUT [Invitrogen]) and incubated on a rotator for 1 h at 4°C. The lysate was cleared by centrifuging at 27,000 × *g* for 20 min at 4°C. Cleared lysate was incubated with anti-FLAG antibody-coated magnetic agarose beads (Pierce) for 4 h at 4°C. The beads were then immobilized by magnet and washed four times with RIP buffer. Protein was eluted twice using 50 µL of elution buffer (0.1 M glycine [pH 2]). Elution volumes were separated on 4%–15% Mini-PROTEAN TGX stain-free precast polyacrylamide gels (Bio-Rad) at 150 V. Gels were stained with Coomassie blue, and bands corresponding to Puf4-FLAG were excised. Excised bands were stored at −80°C until trypsin digestion and LC-MS/MS analysis.

*For protein–protein interactions*: Immunoprecipitations using the Puf4-FLAG overexpression strain, Puf4-FLAG 462 RtoK clone 1, Puf4-FLAG 783/785 RtoK clone 2, and H99 cells were performed as described above. Instead of glycine elution, bound proteins were eluted using FLAG elution buffer (25 mM HEPES-KOH [pH 7.9], 2 mM MgCl_2_, 20% glycerol, 300 mM KCl, 1× cOmplete protease inhibitor (Roche), 1× PhosSTOP phosphatase inhibitor, and 0.5 mg/mL 3× FLAG peptide). After washing four times with RIP buffer (15-min each), the beads were eluted three times using 100 µL of FLAG elution buffer. Elution volumes were combined and stored at −80°C until trypsin digestion and LC-MS/MS analysis. Prior to LC-MS/MS analysis, the buffer was exchanged for one without glycerol and compatible with trypsin digestion.

*Protein digestion:* A surfactant-aided precipitation/on-pellet digestion protocol was adopted using our previously published method with slight modification (35). Samples were transferred to 10-kDa MWCO centrifugal filter units (MilliporeSigma) for buffer exchange and concentration. Three consecutive centrifugation steps with the addition of 200, 400, and 400 μL 50 mM Tris-formic acid (FA) (pH 8.4) were performed at 14,000 × *g* at room temperature for 20 min, and samples were concentrated to a final volume of 20 μL. A total of 60 μl 0.5% SDS was then added to each sample, and the filter units were vortexed vigorously for 10 min. Concentrated samples were transferred to LoBind microcentrifuge tubes (Eppendorf). Proteins were sequentially reduced by 10 mM dithiothreitol at 56°C for 30 min and alkylated by 25 mM iodoacetamide at 37°C in darkness for 30 min. Both steps were performed in a thermomixer (Eppendorf) with rigorous shaking. Proteins were then precipitated by adding 6 volumes of chilled acetone with vortexing, and the mixture was incubated at −20°C for 3 h. Samples were then centrifuged at 20,000 × *g* at 4°C for 30 min, and the supernatant was removed. Protein pellets were gently rinsed with 500 μL methanol, air-dried for 1 min, and resuspended in 46 μL 50 mM Tris-FA (pH 8.4). A total volume of 4 μL trypsin (Sigma Aldrich), reconstituted in 50 mM Tris-FA (pH 8.4) to a final concentration of 0.25 μg/μL, was added for a 6-h tryptic digestion at 37°C with constant shaking in a thermomixer. Digestion was terminated by adding 0.5 μL FA, and samples were centrifuged at 20,000 × *g* at 4°C for 30 min. The supernatants were carefully transferred to LC vials for analysis.

*LC-MS analysis:* The LC-MS system consists of a Dionex UltiMate 3000 nano-LC system, a Dinoex UltiMate 3000 micro-LC system with a WPS-3000 autosampler, and an Orbitrap Fusion Lumos mass spectrometer. A large-inner diameter (i.d.) trapping column (300-µm i.d. × 5 mm) was implemented before the separation column (75-µm i.d. × 65 cm, packed with 2.5-µm XSelect CSH C_18_ material) for high-capacity sample loading, cleanup, and delivery. For each sample, 12 µL of derived peptides was injected twice consecutively for LC-MS analysis. Mobile phase A and B were 0.1% FA in 2% acetonitrile and 0.1% FA in 88% acetonitrile, respectively. The 180-min LC gradient profile was 4% phase B for 3 min, 4%–9% phase B for 5 min, 9%–31% phase B for 117 min, 31%–50% phase B for 10 min, 50%–97% phase B for 1 min, 97% phase B for 17 min, and then equilibrated to 4% phase B for 27 min. The mass spectrometer was operated under data-dependent acquisition mode with a maximal duty cycle of 3 s. MS1 spectra were acquired by Orbitrap under 120k resolution for ions within the *m/z* range of 400–1,500. Automatic gain control and maximal injection time were set at 175% and 50 ms, respectively, and dynamic exclusion was set at 60 ± 10 ppm. Precursor ions were isolated by quadrupole using a *m/z* window of 1.6 Th and were fragmented by high-energy collision dissociation. MS2 spectra of a precursor ion fragmented were acquired by a back-to-back Orbitrap and Ion Trap scheme. Orbitrap was operated under 15 k resolution with an automatic gain target target of 100% and a maximal injection time of 35 ms; Ion Trap was operated under rapid scan rate with an automatic gain control target of 100% and a maximal injection time of 50 ms. Detailed LC-MS settings and relevant information can be found in a previous publication by Shen et al. (36).

*Data processing:* To identify the Puf4 interactome and characterize PTMs of Puf4, two separate sets of data processing procedures were applied to the LC-MS files. For interactome identification, LC-MS files were searched against *Cryptococcus neoformans* var. *grubii* serotype A Swiss-Prot+TrEMBL protein sequence database (7,429 entries) using Sequest HT embedded in Proteome Discoverer 1.4 (Thermo Fisher Scientific). A target-decoy approach using a concatenated forward and reverse protein sequence database was applied for false-discovery rate estimation and control. The searching parameters included (i) precursor ion mass tolerance, 20 ppm; (ii) product ion mass tolerance, 0.8 Da; (iii) maximal missed cleavages per peptide, 2; (iv) fixed modifications, cysteine carbamidomethylation; (v) dynamic modifications, methionine oxidation and peptide N-terminal acetylation. Search result merging, protein inference/grouping, and false-discovery rate control were performed in Scaffold 5 (Proteome Software, Inc.). For identification, the global protein/peptide false-discovery rate was set to 1.0%, and at least two unique peptides were required for each protein. For quantification, protein abundance was determined by total spectrum counts and total MS2 ion intensities. Results were exported and manually curated in Microsoft Excel.

For Puf4 PTM characterization, LC-MS files were searched against *Cryptococcus neoformans* Puf4 sequence (UniProt protein accession J9VI91) using the same search engine settings with the following additional parameters. For Puf4 methylation, arginine (R) methylation and dimethylation were included in dynamic modifications; for Puf4 phosphorylation, serine (S)/threonine (T)/tyrosine (Y) phosphorylation was included in dynamic modifications. Search results were filtered to only retain peptides with medium and high confidence and were exported from Proteome Discoverer. Data curation was performed using a customized R script.

### Spot plate dilution assay

Cells were grown overnight at 30°C in YPD broth. Overnight cultures were washed with sterile distilled water, and the OD_600_ was adjusted to 1 in water. Adjusted cultures were 1:10 serially diluted five times, and 5 µL of each dilution was spotted onto YPD agar plates containing the selected drugs. Agar plates were incubated for 2–3 days at the temperatures indicated in the text and photographed.

### RNA stability time course

RNA extraction and stability analysis were performed as described previously (26). Briefly, mid-log-stage cultures were supplemented with 250 mg/mL of the transcriptional inhibitor 1,10-phenanthroline (Sigma). Then, 5-mL aliquots of each culture were transferred to snap-cap tubes and pelleted every 15 min for 60 min. Fifty microliters RLT buffer supplemented with 1% β-mercaptoethanol was added to each pellet prior to flash freezing in liquid N_2_. Pellets were stored at −80°C until RNA extraction. Cells were lysed by bead beating using glass beads. RNA was extracted from each sample using the RNeasy mini kit (Qiagen) according to the manufacturer’s instructions. RNA was DNase digested on column using the RNase-free DNase kit (Qiagen) or using the Ambion Turbo DNA-free kit (Thermo Fisher Scientific). cDNA for real-time quantitative PCR (RT-qPCR) was synthesized using the Applied Biosystems high-capacity cDNA reverse transcription kit (Thermo Fisher Scientific). Samples were quantified using the second-derivative maximum method and fitted to a standard curve of five 4-fold serial dilutions of cDNA. *GPD1* was used as the normalization gene. Primer sequences were previously published (28).

### Western blot: protein stability and temperature time course

To assess protein stability of Puf4-FLAG and methyl-deficient mutants, cells were grown in YPD broth at 30°C to mid-log phase (OD600 = 0.5–0.6). Cycloheximide was added at 0.1 mg/mL, and aliquots of the culture were harvested and pelleted at *t* = 0, 1 and 2 h. Lysis and protein quantification were performed as previously described (26). To assess Puf4 levels following temperature stress, cells were grown in YPD broth at 30°C to mid-log phase. A 15 mL aliquot was pelleted and flash frozen, and the remaining culture was pelleted and resuspended in pre-warmed 37°C YPD to an OD600 = 0.6. Every 30 min, 15 mL of culture was pelleted and flash frozen, and the OD600 of the remaining culture was adjusted to 0.5 with 37°C YPD to prevent nutrient depletion. Cells were resuspended in 50 µL lysis buffer (50 mM Tris, pH 7.5, 140 mM NaCl, 0.5% Triton X-100, 1 mM DTT, 10 µL/mL HALT protease and phosphatase inhibitor) and lysed by bead beating for 5 min. The lysates were cleared by centrifugation at 20,050 × *g* for 10 min at 4°C. Protein was quantified using the Pierce 660 Protein Assay kit.

For both stability and steady-state experiments, 15 µg of protein per sample was subjected to SDS-PAGE on a 4%–15% Mini-Protean TGX stain-free precast gels (Bio-Rad) at 150 V. Total protein was imaged using the stain-free gel setting on a BioRad GelDoc XR+. Protein within the gel was then transferred to a nitrocellulose membrane and then blocked with Milk with 5% Tris-buffered saline-Tween 20 (Milk-5%TBS-T) for 1 h, or with BioRad Everyblot Buffer. The blots were then incubated overnight with mouse anti-FLAG antibody (1:1,000 in Milk-5%TBS-T or Everyblot) at 4°C. Next, blots were washed three times with TBS-T for 15 min per wash. Blots were then incubated with anti-mouse IgG-HRP (1;10,000 in Milk-5%TBS-T) for 1 h at room temperature.

### Melanin assay

Melanization agar plates were prepared using asparagine medium (1 g/l asparagine, 0.1 g/l MgSO_4_, 10 mM Na_3_PO_4_, pH 6.5) and 2% agar supplemented with 0.1% glucose and 2 mM 3,4-dihydroxy-l-phenylalanine (L-DOPA) at pH 6.5. Cells were grown overnight in YPD broth at 30°C, pelleted and washed in sterile deionized water after which 10 mL of cells at an OD_600_ of 1.0 in water was pelleted and the supernatant removed. Five microliters of pellet slurry was spotted onto melanin agar plates, incubated overnight at 30°C, and photographed.

### *LAC1* expression and RNA stability analysis

Cells were grown in YPD broth at 30°C to mid-log phase and centrifuged, and pellets were resuspended in asparagine medium without glucose (1 g/liter asparagine, 0.1 g/liter MgSO4, 10 mM 10mM Na_3_PO_4_, pH 6.5). Cells were incubated for 4 h at 30°C. Aliquots were collected every hour, and pellets were flash frozen and stored at −80°C. RNA extraction and RT-qPCR were performed as described above. For *LAC1* RNA stability experiments, overnight cultures grown in YPD broth were used to start cultures in asparagine medium without glucose at OD600 of 0.5 and incubated at 30°C for 4 h. Cultures were then treated with the transcriptional inhibitor 1,10-phenanthroline, and RNA stability was determined as described in the RNA stability time course section.

### Quantification and statistical analysis

For RNA stability analyses, statistical differences of decay rates were compared by determining the least-squares fit of one-phase exponential decay nonlinear regression analysis with GraphPad Prism software. Significance between curves was detected with a *P* value cutoff of 0.05, which determined that the data from two different curves create different regression lines, therefore, yielding different half-lives of the same transcript investigated in different mutants. All error bars throughout the article show standard error of the mean (SEM) unless otherwise noted.

## RESULTS

### Arginine methylation in *C. neoformans* Puf4 and evolutionary divergence of RG domains

**Fig 1:**
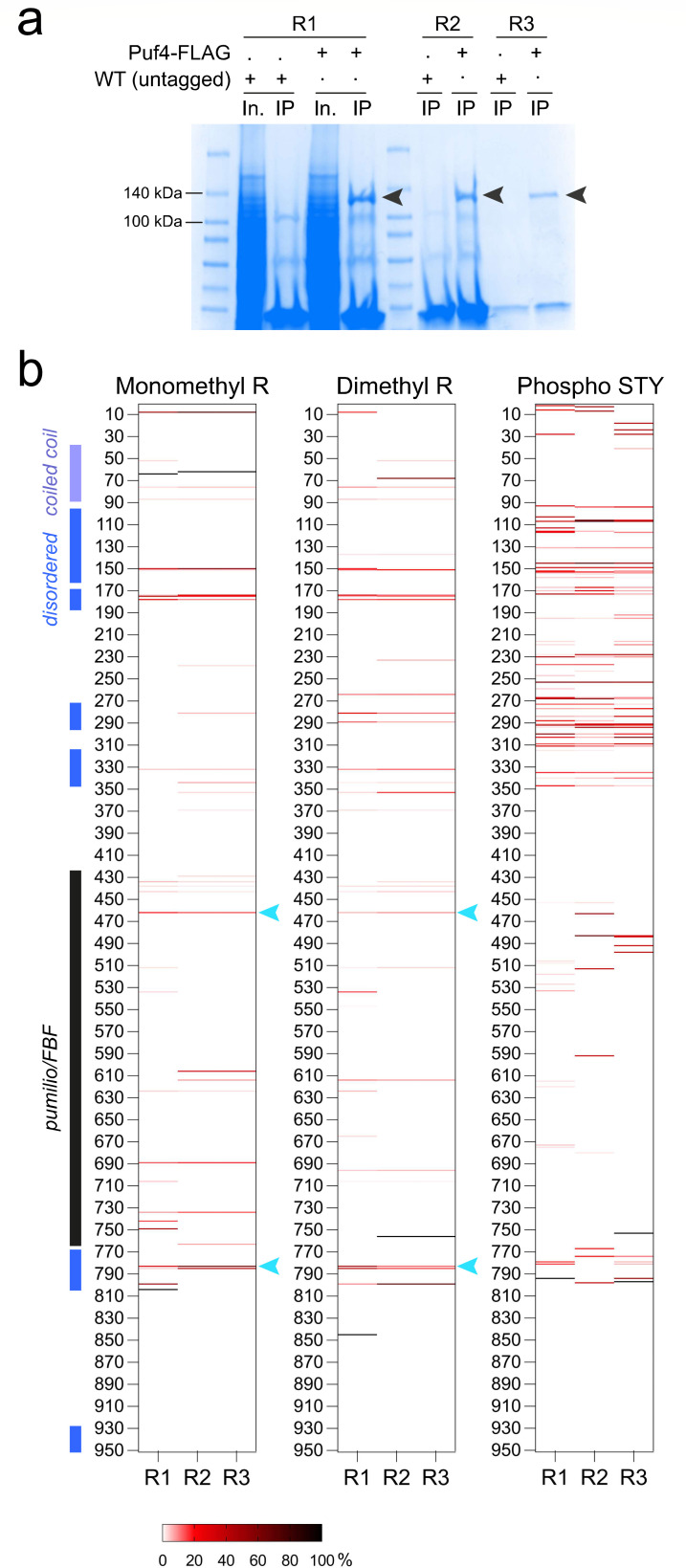
Post-translational modifications of *C. neoformans* Puf4—arginine methylation and phosphorylation. (**a**) Immunoprecipitation of Puf4. Coomassie-stained gel shows three replicates of immunoprecipitated Puf4-FLAG. Arrowheads show the Puf4-FLAG bands that were excised and analyzed for PTMs using label-free LC-MS/MS. (**b**) PTM landscape of Puf4. Heatmaps show the percentage of modifications at each amino acid site for monomethyl arginine methylation, dimethyl arginine methylation, and STY phosphorylation. Percentage of modifications for each amino acid was calculated by dividing the number of modifications at one site by the number of times that site was detected among all peptides. Important protein domains are noted on the left along with the amino acid positions.

To characterize the PTM landscape of Puf4, we performed label-free liquid chromatography-tandem mass spectrometry (LC-MS/MS) of Puf4 immunoprecipitated from lysates of a *C. neoformans* strain overexpressing Puf4 at 30°C (Fig. 1a, bands excised from the gel are indicated with arrowheads). Specifically, we mapped the monomethyl and dimethyl arginine modifications across the protein sequence (Fig. 1b). Because Puf4 is downstream in the calcineurin pathway and is dephosphorylated by the phosphatase Cna1 (37), we also mapped the phosphorylation of serine/threonine/tyrosine (STY) residues. This approach yielded 81.05%, 74.50%, and 74.43% Puf4 sequence coverage in three biological replicates. The percentage of modifications per amino acid site was calculated by dividing the number of times a site was modified by the total number of times the same site was detected across sampled tryptic peptides. The results revealed that *C. neoformans* Puf4 is methylated and phosphorylated under basal conditions (Fig. 1b). The methyl marks were distributed throughout the protein, but there was strong methylation of arginines in the disordered domains, with high confidence across the three biological replicates. By contrast, very few of the arginine residues in the PUF domain were methylated, suggesting that these sites are important for critical interactions and may be inaccessible. The phosphorylation of STY residues was similarly concentrated in the disordered domains (Fig. 1b). Thus, both arginine methylation and STY phosphorylation in Puf4 likely influence RBP function as well as protein–protein or protein–RNA interactions.

**Fig 2:**
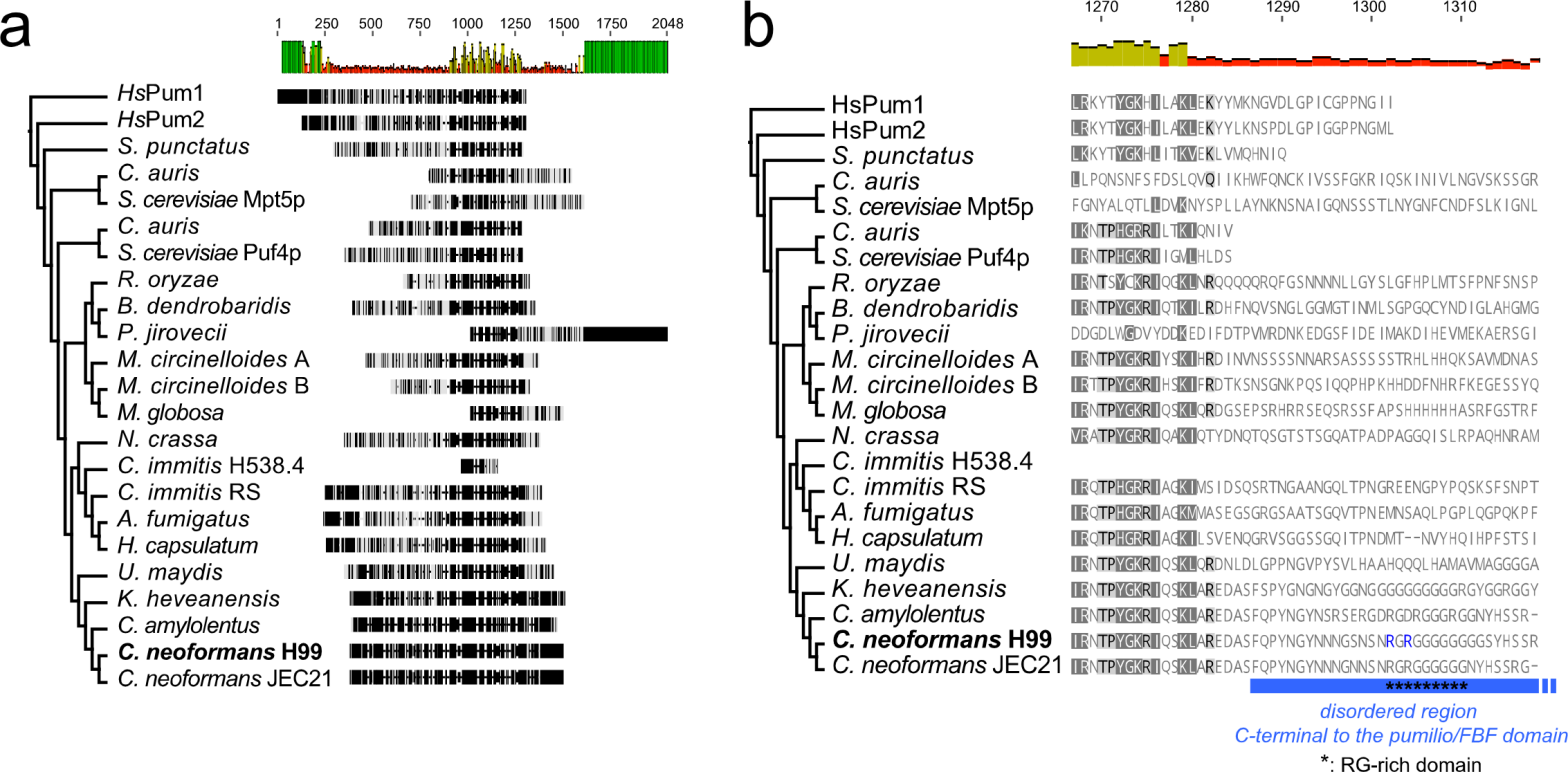
Evolutionary divergence of Puf4 and an RG-rich domain. (**a**) Phylogenetic analysis of Puf4 across fungi. Protein sequences used for analysis were exported from NCBI (IDs are compiled in Table S1), and phylogenetic analysis was performed using default settings on Geneious. Human Pum1 and Pum2 are included as outgroups. Bars above show the conservation of each site. Yellow bars in the middle correspond to the highly conserved Pumilio domain repeats. Numbers above represent the amino acid positions in alignment. (**b**) Evolutionary divergence of the RG-rich domain. The blue box highlights the disordered domain located C-terminal to the Pumilio domain. Asterisks highlight the RG-rich methylation hub located within this disordered domain, which is unique to *C. neoformans*.

To investigate the evolutionary conservation of critical domains in Puf4 across the fungal kingdom, we performed a phylogenetic analysis including fungi representing different taxonomic divisions (Fig. 2a). Analysis revealed that the PUF domain is highly conserved in fungi, but disordered domains and RG-rich domains are more divergent (Fig. 2b, RG-rich domain is shown by the asterisks within the disordered domain which is highlighted by the blue box. The species-specific RG-rich domain C-terminal to the PUF domain (i.e., the methylation hub) that we identified in our phylogenetic analyses exhibited both monomethyl and dimethyl modifications (Fig. 1b). The proximity of this methylation hub to the RNA-binding domain and its presence within a disordered domain suggest that arginine methylation likely regulates protein function. Lack of conservation in the RG-rich domain suggests a divergence in Puf4 function because disordered domains evolve faster and are important for intermolecular interactions and liquid–liquid phase separation (15, 38). The RG-rich domains appeared to be unique to each species (Fig. 2b) and, thus, could either be post-translationally modifiable domains that are not under constant selection or have species-specific roles.

### Mutations for methyl deficiency of select residues do not impact the complementation of most Puf4 deletion phenotypes

**Fig 3:**
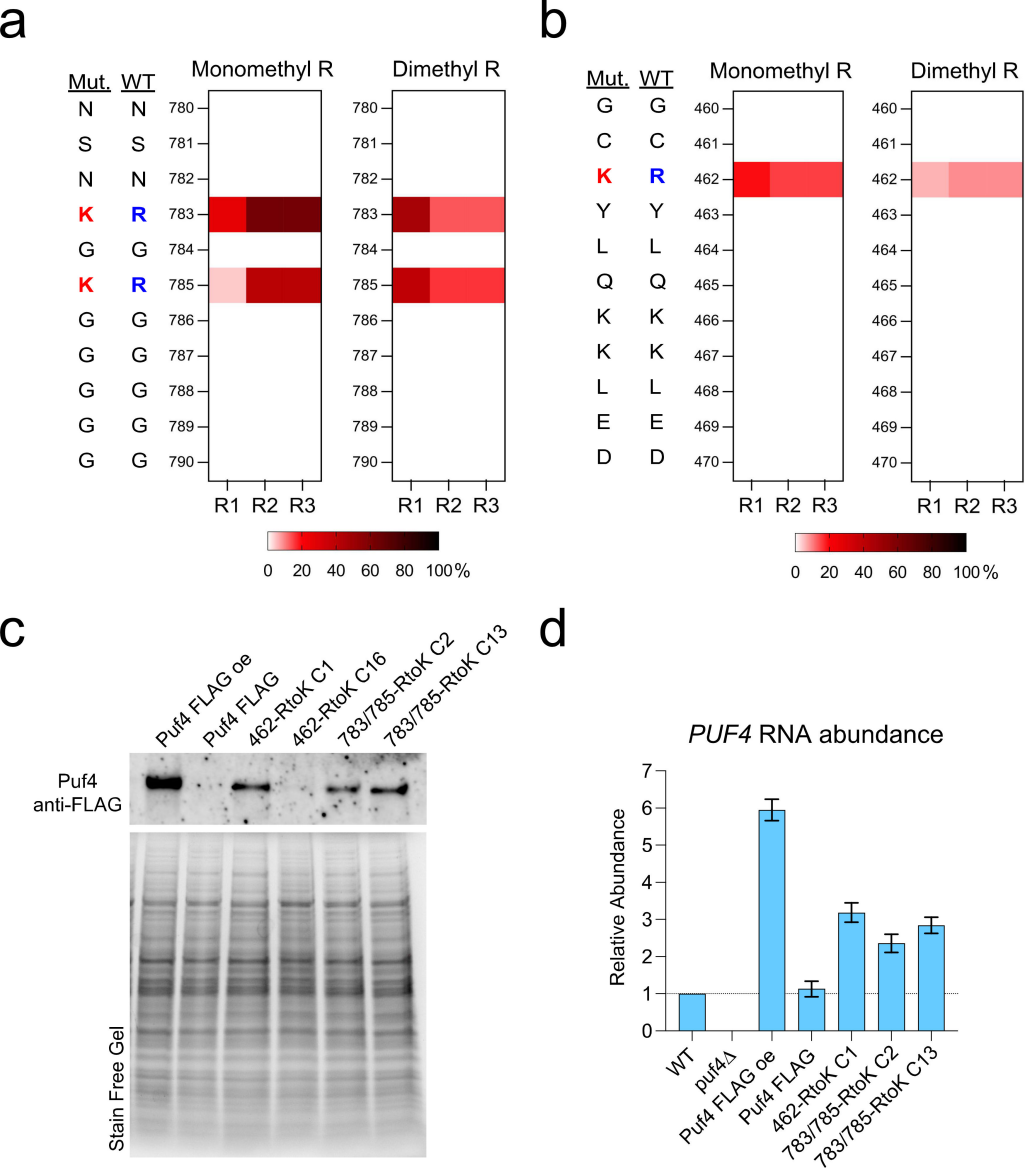
Selection of methylated residues and expression of methyl-deficient mutants in the *puf4*△ background. (**a and b**) Arginine methylation of the RG-rich methylation hub located within the disordered domain C-terminal to the Pumilio domain (Arg783 and Arg785), and a site located within the Pumilio domain (Arg462) are shown in detail. (**c**) Western blot analysis of methyl-deficient FLAG-tagged complementation strains compared to the single-copy Puf4-FLAG complement and the multi-copy Puf4-FLAG overexpression strains detected by anti-FLAG monoclonal antibody. Stain-free gel is included as a loading control. (**d**) Quantification of *PUF4* mRNA expression in the methyl-deficient mutant-expressing strains relative to the wild type and Puf4-FLAG expressing strains. Three biological replicates (except for *puf4*∆, which has 2) and two technical replicates per biological replicate were plotted.

We picked two highly modified residues to further probe the PTM–function relationship: Arg783/Arg785, located within the RG-rich methylation hub C-terminally adjacent to the PUF domain (Fig. 3a), and Arg462, located within the PUF domain (Fig. 3b). We mutated these arginines to lysines (RtoK) on a plasmid expressing Puf4-FLAG to then establish cell lines expressing FLAG-tagged methyl-deficient Puf4 mutants in *puf4*∆ background. We evaluated FLAG-tagged wild-type and methyl-deficient Puf4 strains for complementation of the *puf4*△ mutant phenotypes.

We established two independent complement strains for each mutation and assessed for protein expression by western blot (Fig. 3c). One challenge we faced was that a single copy Puf4-FLAG expression strain was near the limit of detection by western blot, but fully complemented Puf4 phenotypes. We compared the methyl-deficient mutant expressing strains with a multicopy expresser of Puf4-FLAG that is detectable by western blot. We were unable to detect expression of Puf4-FLAG 462 RtoK clone 16 by western blot, and therefore, we excluded it from further analysis. Additionally, *PUF4* mRNA levels of the remaining three methyl-deficient expressing strains are quantified in Fig. 3d.

**Fig 4:**
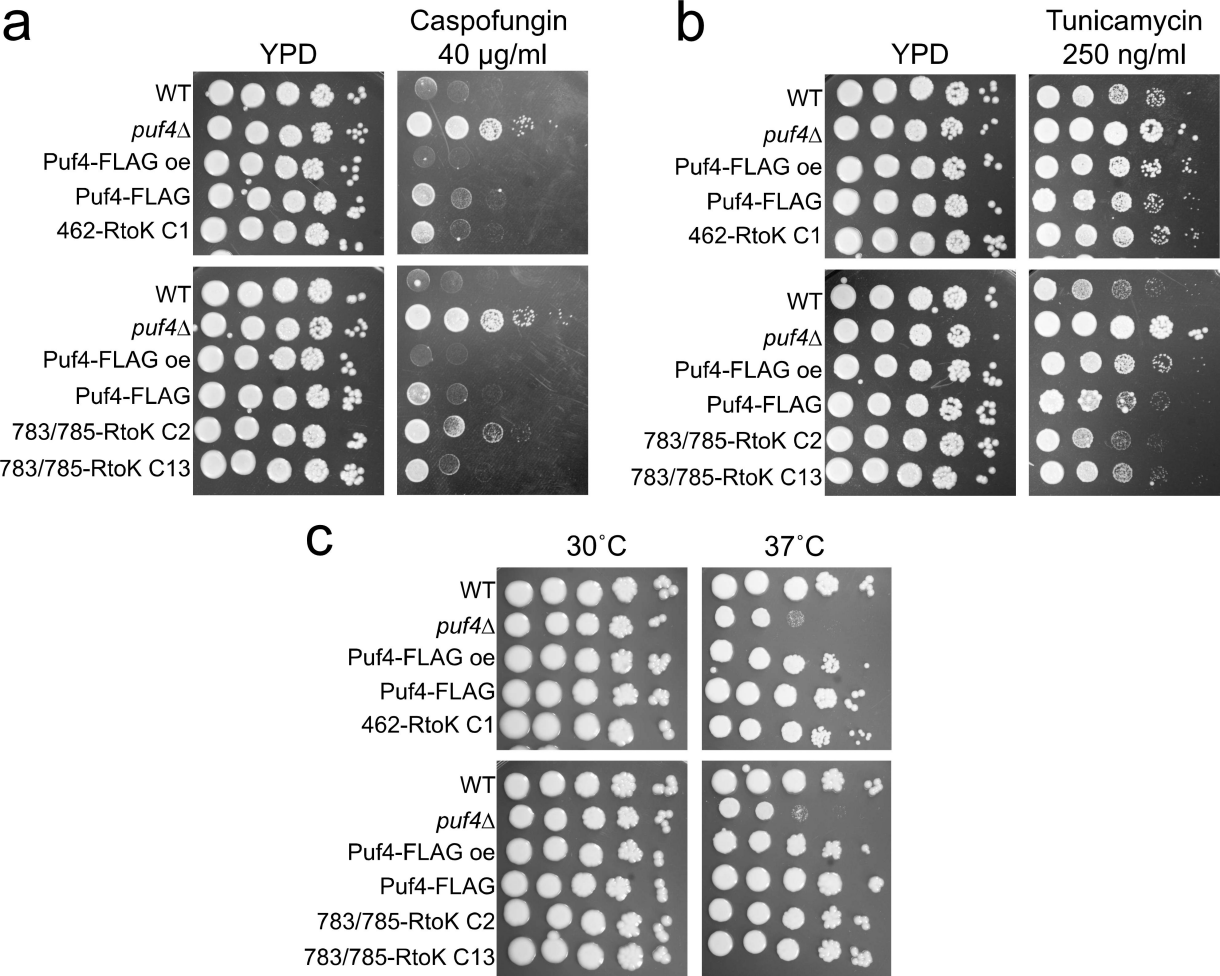
Methyl-deficient mutants of Puf4 complement *puf4*△ cell wall, ER stress, and thermal stress sensitivity. (**a**) Complementation of *puf4*△ caspofungin resistance by expression of 462 RtoK Puf4-FLAG and 783/783 RtoK Puf4-FLAG. (**b**) Complementation of *puf4*△ tunicamycin resistance by expression of 462 RtoK Puf4-FLAG and 783/783 RtoK Puf4-FLAG. (**c**) Complementation of *puf4*△ temperature sensitivity by expression of 462 RtoK Puf4-FLAG and 783/783 RtoK Puf4-FLAG.

Our previous work has implicated Puf4 in regulating ER stress, caspofungin resistance, and cell wall stress (26, 27). To determine if methyl-deficient mutations at either Arg462 or Arg783/Arg785 would abrogate the ability of Puf4 to promote cell wall stress and ER stress adaptation, we compared the sensitivity of the mutants to that of the wild type and complementation strains to caspofungin and tunicamycin, respectively. We found that both mutant Puf4 alleles were able to suppress the caspofungin resistance of the *puf4*△ mutant to an extent similar to that of the single-copy complement, but not quite to the level of wild type (Fig. 4a). Similarly, both methyl-deficient mutants suppressed the tunicamycin resistance of the *puf4*△ mutation (Fig. 4b). Because *PUF4* deletion causes a defect in thermotolerance of *C. neoformans*, which is critical for the pathogenicity of the fungus, we investigated the effect of methylated residues on growth at different temperatures. Both methyl-deficient Arg462 and Arg783/Arg785 mutants complemented the thermotolerance defect in the *puf4*△ strain at 37˚C (Fig. 4c). Overall, our results highlight that the identified methylated arginine residues are not critical for the Puf4 phenotypes evaluated herein, which are a downstream output of Puf4 regulation.

**Fig 5:**
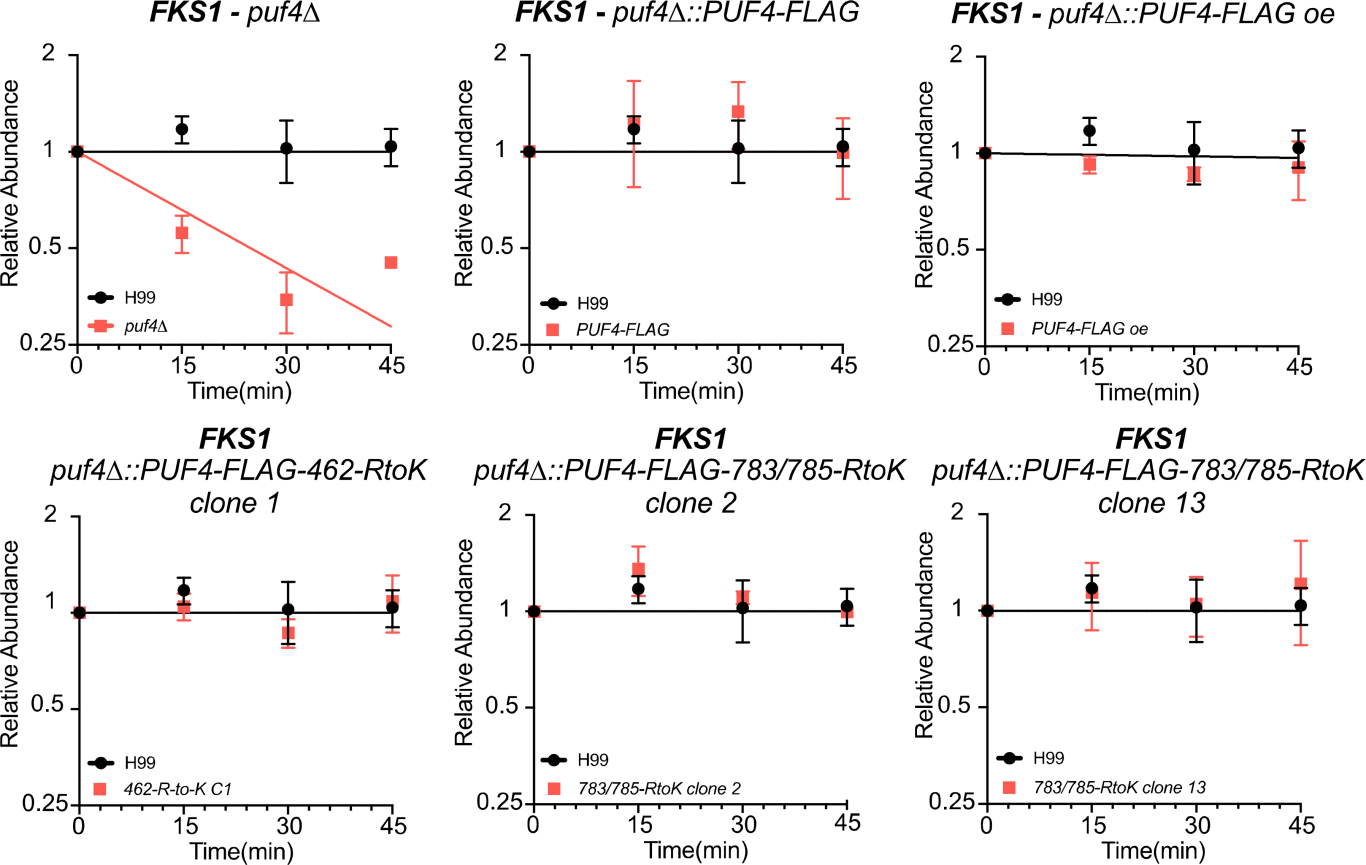
Methyl-deficient mutants of Puf4 complement the *FKS1* mRNA decay phenotype of the *puf4*∆ mutant. *FKS1* mRNA abundance was determined by RT-qPCR following transcription shut-off to determine decay kinetics. Three biological replicates with two technical replicates were plotted. The decay kinetics were calculated using one-phase exponential decay analysis. Expression of 462 RtoK Puf4-FLAG and 783/783 RtoK Puf4-FLAG restores *FKS1* mRNA stability to that of wild type.

To investigate potential regulatory roles for the methylated residues in the direct function of Puf4, we assessed the regulation of mRNA stability of *FKS1*, the target of caspofungin, which is destabilized in the absence of Puf4 (26). Again, we found that each mutant allele was able to suppress the *FKS1* destabilization phenotype of the *puf4*△ mutant (Fig. 5). We previously identified additional mRNAs encoding cell wall remodelers that exhibited Puf4-dependent mRNA stability. We assessed the ability of the methyl-deficient mutants to suppress the destabilization of the *CHS4* mRNA and found that the expression of the methyl-deficient Puf4-FLAG alleles was able to restore a *CHS4* mRNA half-life similar to that of expressing wild-type Puf4 (Fig. S1).

Our previous work on Pum1, another *C. neoformans* PUF family member, demonstrated auto-regulation through 5′ UTR interactions (33). We identified a single canonical Puf4-binding element in the 5′ UTR of the *PUF4* mRNA (Fig. S2a). We next investigated if methylation-deficient mutations would alter the stability of the *PUF4* mRNA. In each case, the *PUF4* mRNA stability was unchanged relative to wild type *C. neoformans*, suggesting that methylation of these sites does not affect the mRNA stability of *PUF4* itself (Fig. S2b). We also assessed protein stability by western blot following translational shutoff with cycloheximide and found that neither methyl-deficient mutation altered the stability of Puf4-FLAG protein at 30°C (Fig. S2c).

### Puf4 deletion and methyl-deficient complement strains have a melanization defect associated with impaired induction of *LAC1* expression

**Fig 6:**
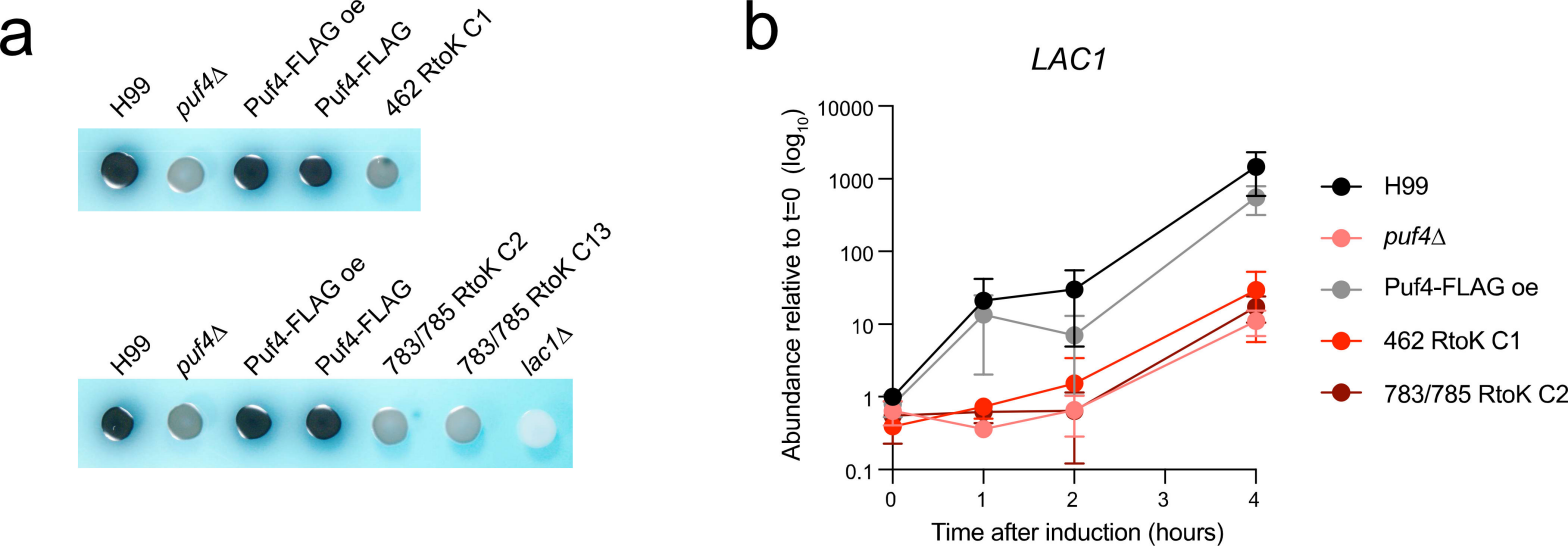
Methyl-deficient mutants of Puf4 fail to complement the melanization defect of the *puf4*△ mutant. (**a**) Melanin formation assay using L-DOPA as substrate. A *lac1*△ mutant is included as a negative control. (**b**) Induction of the *LAC1* gene in response to carbon starvation is defective in the methyl-deficient complementation strains. Cells were collected at the indicated time points following carbon starvation in asparagine media. *LAC1* abundance was determined using RT-qPCR. Three biological replicates with two technical replicates were plotted. Error bars represent the standard deviation.

In our further characterization of the *puf4*△ deletion mutant, we observed that the mutant had an obvious deficiency in melanin production. To determine if the melanin deficiency may depend on methylation at Arg462 or Arg783/Arg785 we assessed complementation by melanin spot assay. The melanin deficiency of *puf4*△ was complemented by single-copy Puf4-FLAG and Puf4-FLAG overexpression (Fig. 6a). However, the methyl-deficient mutants did not complement the melanization defect.

We next examined the strains for expression of *LAC1*, which encodes the multi-copper oxidase laccase that catalyzes the oxidation of l-DOPA to produce melanin (39). *LAC1* expression is induced by glucose withdrawal; thus, we performed a glucose starvation time course experiment over 4 h. The wild-type strain and the Puf4-FLAG overexpression complement strain showed robust induction of *LAC1*, whereas the *puf4*∆ and methyl-deficient mutants did not (Fig. 6b). We also investigated the *LAC1* RNA stability under glucose starvation and showed that the stability of *LAC1* is not dependent on Puf4 and methyl-deficient complement strains do not have altered *LAC1* stability (Fig. S3). This suggests that the inability to induce *LAC1* expression underlies the observed melanization defect in the methyl-deficient mutants. While methyl-deficient mutants successfully complement the Puf4-deletion phenotypes that are associated with mRNA decay such as caspofungin resistance and ER stress, they do not complement the laccase phenotype which stems from lack of transcriptional induction. This implies that arginine methylation may be a mechanism to separate transcriptional (either directly or through regulation of transcription factors) and post-transcriptional roles of Puf4 in gene expression regulation and stress adaptation.

### Methyl-deficient mutants have unique and altered protein–protein interactions

To further evaluate the role of Puf4 in orchestrating various gene regulatory events, we investigated the protein interaction networks of Puf4 at 30°C and 37°C. Because the *puf4*∆ mutant has a defect in thermotolerance, we reasoned that we would be able to discern temperature-specific protein interaction networks. We immunoprecipitated Puf4 from the Puf4-FLAG overexpression strain and the methyl-deficient strains (Puf4-FLAG 462 RtoK clone 1 and Puf4-FLAG 783/785 RtoK clone 2) and identified interacting proteins via MS. We used stringent criteria for our MS data filtering and only considered proteins with at least three spectral counts and twofold enrichment over that in an untagged mock immunoprecipitation. Pearson correlation coefficient of counts between two biological replicates ranged from 0.88 to 0.97 (Fig. S4). Interactions that were captured in only a single biological replicate were excluded.

**Fig 7:**
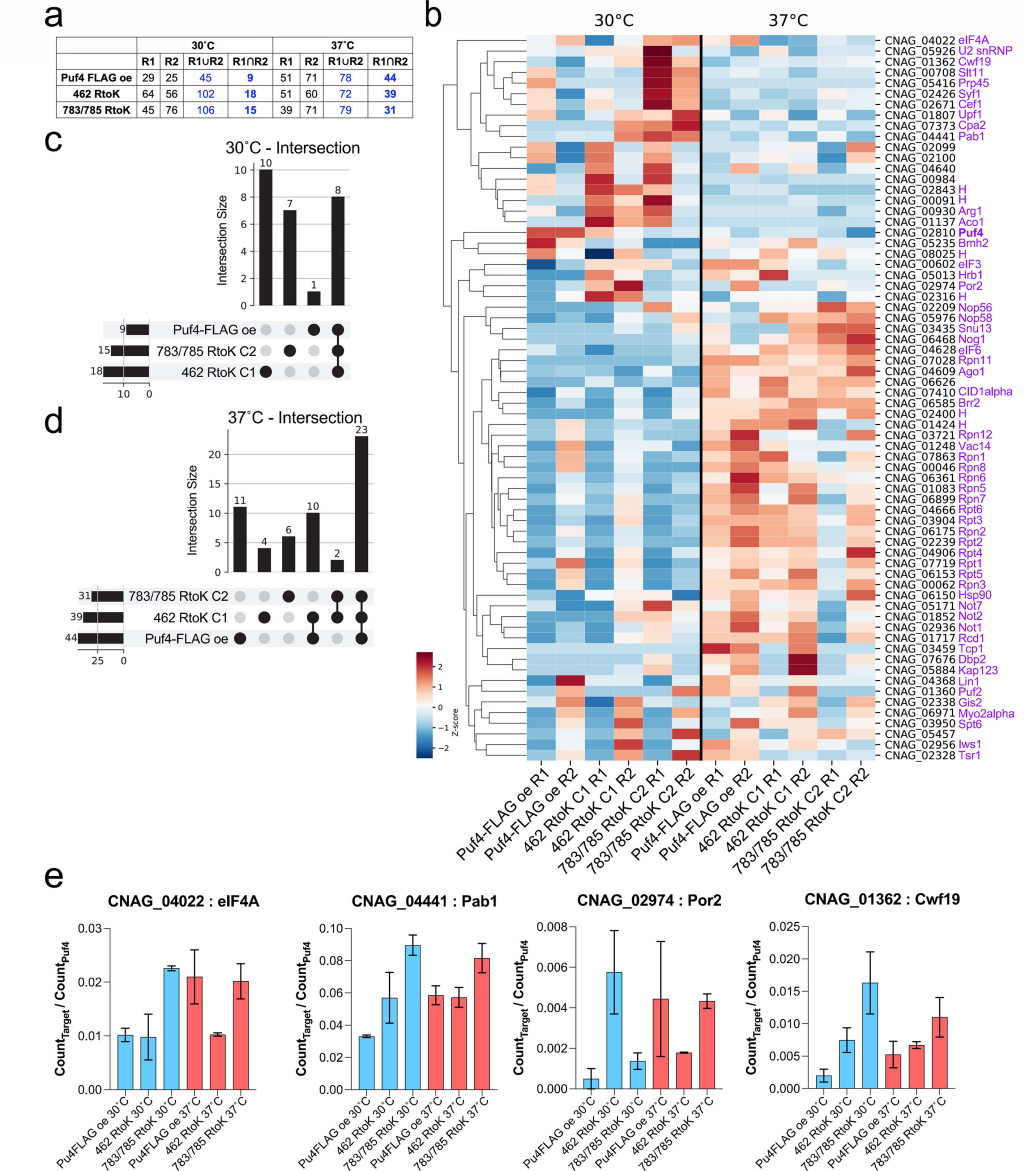
Methyl-deficient mutants have unique and altered protein–protein interactions. (**a**) Summary table. Puf4-FLAG overexpression strain and methyl-deficient mutants were grown at 30°C or 37°C in YPD broth. FLAG immunoprecipitation was performed to capture protein–protein interactions, and interacting proteins were identified using LC-MS/MS in two biological replicates. Untagged cells were used to detect nonspecific interactions. Interacting proteins with at least three spectral counts twofold over the background amount were selected for each replicate. R1∪R2 column shows the number of unique proteins that are in at least one of two replicates. R1∩R2 column shows the number of proteins that were common between two replicates. (**b**) Heat map of row normalized spectral counts for protein interactions identified in both replicates (R1∩R2). (**c**) Upset plot of protein interactions identified in both replicates at 30°C. (**d**) Upset plot of protein interactions identified in both replicates at 37°C. (**e**) Relative enrichment of selected proteins across strains and temperatures.

**Fig 8:**
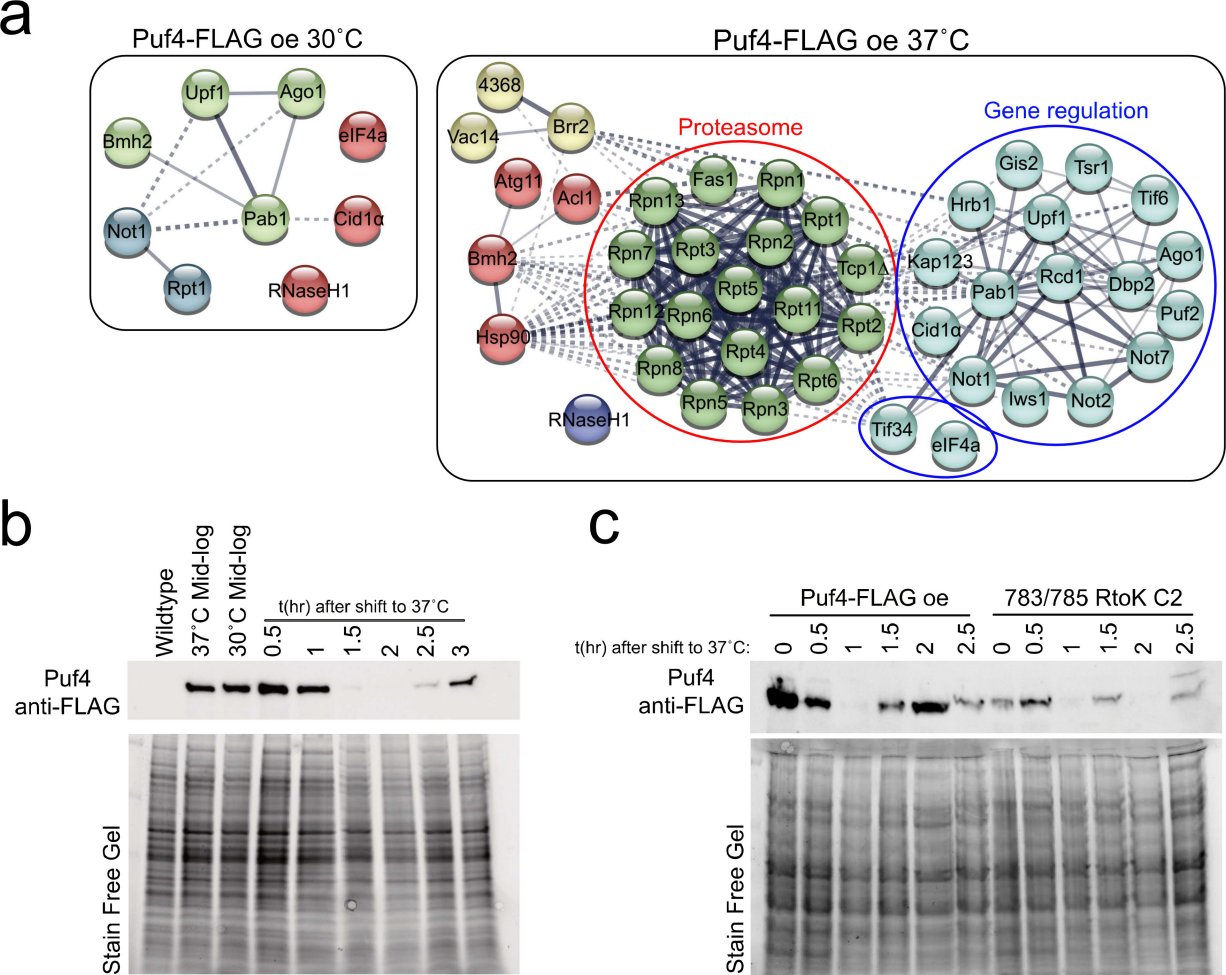
Puf4 interactions reveal connections to the proteasome and mRNA decay pathways. (**a**) STRING analysis of Puf4-FLAG interactions reveal connections to the proteasome and mRNA decay pathways. (**b and c**) Puf4-FLAG protein is transiently repressed in response to thermal stress independent of Arg783/Arg785. Cells growing at 30°C were transitioned to 37°C and Puf4-FLAG (either wild-type overexpression or methyl-mutant) levels were probed by western blotting.

Our results revealed that Puf4 has temperature-specific protein–protein interactions (Fig. 7a and b). Puf4 interacted with Ago1 (CNAG_04609; argonaute-1), Upf1 (CNAG_01807; nonsense-mediated decay helicase), eIF4A (CNAG_04022; translation initiation factor), Not1 (CNAG_02936; Ccr4-Not complex subunit), Pab1 [CNAG_04441; poly(A) binding protein], Cid1 [CNAG_07410; poly(A) polymerase], Bmh2 (CNAG_05235; 14-3-3 domain-containing protein), RNaseH1-domain containing protein (CNAG_08025), and Rpt1 (CNAG_07719; proteasome subunit) at 30°C (Fig. 7b and 8a). Puf4 interacted with a more diverse set of proteins at 37°C, including Lin1 (CNAG_04368; U5 snRNP complex protein), Iws1 (CNAG_02956; transcription factor), Kap123 (CNAG_05884; importin), Dbp2 (CNAG_07676, pre-mRNA splicing protein), and Hsp90-like protein (CNAG_06150; heat shock chaperone) as well as 13 subunits of the proteasome (Fig. 7b and 8a). These results suggest that Puf4 interacts with proteins involved in cytoplasmic processes (such as Not1) at 30°C but begins to interact with proteins involved in nuclear RNA processes as well as the proteasome complex at higher temperatures.

We identified 26S proteasome regulatory subunit 7 (CNAG_07719; *S. cerevisiae* Rpt1) as the single protein unique to the interactome of Puf4-FLAG at 30˚C (Fig. 7c). *S. cerevisiae* Rpt1 is an essential gene and is a multifunctional protein involved in protein catabolism as well as RNA polymerase II preinitiation complex assembly (40). Ten proteins were unique to the interactome of Puf4-FLAG 462 RtoK at 30˚C these included mitochondrial proteins, and a cytoplasmic protein involved in glucose metabolism. Puf4-FLAG 783/785 RtoK had seven unique interactions, but four were included in one replicate of Puf4-FLAG at 30˚C (Fig. 7c). The three interactions that we can report with confidence included Cpa2 (CNAG_07373; carbamoyl-phosphate synthase) and Rcd1 (CNAG_01717; cell differentiation protein). At 37˚C, four proteins were found to interact with Puf4-FLAG 462 RtoK (Fig. 7d), but only one of these was not present in a replicate of a different strain: myosin class V heavy chain (CNAG_06971). The five unique high-confidence interactions with Puf4-FLAG 783/785 RtoK at 37°C were nuclear proteins. These findings suggest that Puf4 has temperature- and methylation-dependent protein interaction networks. A less stringent analysis of the interactome intersections demonstrating hits that appeared in at least one replicate is shown in Fig. S5.

We then calculated the prey (target protein) and bait (Puf4-FLAG) ratios from IP/MS spectral counts to semi-quantitatively investigate the binding patterns across methyl-deficient mutants at 30°C and 37°C relative to the abundance of peptides that map to Puf4 (Fig. 7e). We found that overall Puf4 interacts with a higher counts of target proteins at 37˚C compared to 30°C. The Puf4-FLAG 783/785 RtoK mutant showed a greater enrichment of peptides that mapped to eIF4A and Pab1 compared to wild-type at 30°C. The Puf4-FLAG 462 RtoK mutant had a fewer peptides that mapped to eIF4A at 37°C while both wild-type and Puf4-FLAG 783/785 RtoK mutant had greater enrichments. Additionally, a mitochondrial protein (Por2) and a nuclear protein (Cfw19) showed altered trends suggesting that Puf4 may have distinct roles and interacting partners at different cellular compartments. These trends suggest that methyl-deficient mutants may possess different binding affinities to target proteins, and this difference may cause Puf4 to be more immaturely present or absent from complexes involved in critical translation and post-transcriptional regulation. Further binding assays are needed to confirm these hypotheses.

To visualize relatedness in the Puf4-FLAG interactome, we performed STRING analysis (Fig. 8a) (41). The Puf4-FLAG interaction network at 30°C exhibits few interactors with the connections identified related to mRNA decay pathways including nonsense-mediated decay (Upf1), RNA silencing (Ago1), and deadenylation-dependent decay (Not1). In addition, both Pab1 and eIF4A function to promote translation of mRNAs. At 37°C, the STRING analysis revealed higher connectedness with additional post-transcriptional regulators including other RNA binding proteins such as Gis2 and Puf2, and additional components of the Ccr4-NOT complex. Most interesting was the robust association of Puf4 with the proteasome at 37°C. To probe the outcome of this proteasome interaction, we assessed steady-state abundance of Puf4-FLAG protein in a time course following a shift from 30°C to 37°C. We found that Puf4-FLAG protein abundance was rapidly and transiently reduced in response to thermal stress (Fig. 8b). Because the proteasome interactions were retained in the interactome data when Arg562 and Arg783/Arg785 were mutated, we expected the repression of Puf4 to be retained in the methyl-deficient mutants. As expected, Puf4-FLAG 783/785 RtoK was repressed in response to thermal stress. Future work is needed to determine the region of Puf4 that is required for its repression, and to investigate post-translational modifications that influence this repression.

## DISCUSSION

Although the PUF domains in RBPs are evolutionarily conserved, we found that the RG-rich domains, which may serve as methylation hubs, are not in Puf4. Mutations that interfered with arginine methylation at specific residues in Puf4 did not grossly impact Puf4-related phenotypes except for melanization and some protein-protein interactions. Our data suggest that the PTMs in the RG-rich domain of Puf4 may fine-tune the protein–protein interactions that regulate various cellular processes (26, 27).

The identification of methylation-dependent roles for Puf4 in cellular processes important for *C. neoformans* alludes to PTM-driven unique functional states. For example, we observed differential protein-protein interactions by the two methyl-deficient mutants. PTM-driven interactions may alter protein fitness and functional range. The concept of PTM-driven functional diversity may explain why deletion of a gene that regulates essential cellular processes such as ribosome biogenesis and translation does not always produce a drastic phenotype. Multiple subtly different PTM-driven functional states may neutralize or balance each other, effects that are masked by full deletion of the gene. Further biochemical and cellular assays using various arginine mutants and recombinant proteins that would skew protein functional range are necessary to further explore this hypothesis.

The inability of the methyl-deficient mutants to complement the melanization defect of the *puf4*△ strain suggests that the modification of arginines in Puf4 can drive function. The partition of function was supported in our interactome analysis, in which the 783/785 mutant interacted with proteins with nuclear function. Given that the melanization defect may stem from a defect in transcriptional induction, it is possible that methylation of Puf4 may impact subcellular localization either driven by specific interactions or resulting in specific interactions.

Puf4 is extensively methylated, so our approach risked choosing specific methyl marks based on uniqueness to the *C. neoformans* protein as with Arg783/Arg785, or location within the RNA binding domain as with Arg462. PTM-function studies focusing on methylated residues located within disordered or coiled-coil domains may further dissect the contribution of methylation on protein functional diversity. A parallel approach to investigating arginine methylation is to investigate the catalyzing enzymes, the protein arginine methyltransferases. This is especially intriguing because *RMT5* (which interacts with Puf4) is the only *C. neoformans* arginine methyltransferase for which deletion similarly reduces thermotolerance at elevated temperatures (25). Thus, we speculate that Rmt5 acts on Puf4 to establish thermotolerance. Future work will explore the Puf4 residues that are targeted by Rmt5.

Our analyses of protein networks revealed temperature-dependent protein–protein interactions with the wild-type Puf4 and its methyl-deficient mutants. Of note, we found that Puf4 interacts with Ccr4/Not and proteasome complexes, which suggests that it is a central regulator of mRNAs and proteins marked for degradation, perhaps as a mitigator of cellular stress or damage. Our analyses also revealed putative methylation-dependent interactions or methylation-dependent changes in binding patterns as suggested by prey/bait ratios. Future work is necessary to validate the protein-protein interactions using orthogonal approaches. Some of these interactions may be mediated by PTM cross talk, as our results suggest there is cross talk involving different methylation sites and between sites of methylation and phosphorylation. Indeed, PTM cross talk is an important aspect of functional regulation and diversity (16, 42–44). One example of PTM cross talk is the dynamic regulation of chromatin states through histone H3 acetylation and phosphorylation (45). Because Puf4 is a downstream target of calcineurin and dephosphorylated by Cna1, future studies exploring PTM cross talk may be necessary to uncover the PTM regulatory mechanisms of this important RBP.

The interaction of Puf4 with the proteasome at 37°C coupled with the observed rapid repression of Puf4 is consistent with our previous hypothesis that repression of Puf4 yields a state that is necessary for stress response. Similarly, our previous work revealed that pharmacological stress induced by Caspofungin causes a drastic decrease in Puf4 protein levels which, in turn, yields increased cell wall chitin content at 30°C, a phenomenon necessary to resist Caspofungin and normally observed at 37°C instead of 30°C (26). Noting that the repression of Puf4 at 37°C is transient suggests that while repression of Puf4 may be required for the initial response during shift from 30°C to 37°C, Puf4 protein rebound may be required to support constitutive growth at 37°C, hence the temperature-sensitive phenotype of the *puf4*∆ mutant.

Although our approach to characterize the PTM landscape of Puf4 yielded high protein coverage and we were confidently able to identify methyl modifications to arginines, we were unable to distinguish between symmetric and asymmetric dimethylation, which can determine how the residue interacts with a “reader” of PTMs. Future studies will include MS approaches that can more confidently detect these differences (reviewed 46). The use of RtoK mutations to dissect the functional importance of modified arginine residues enabled us to conclude that the presence of lysine instead of arginine can change protein function, but we cannot definitively state that the effect is the result of a lack of arginine methylation. This remains to be a major challenge in the field of PTM biology.

The outlined studies and findings in this article explore the gene regulatory network of Puf4 comprehensively through a lens of protein arginine methylation. These results will serve as the basis of future investigations on PTM-driven post-transcriptional events in *C. neoformans*.

## Data Availability

Data tables for MS experiments are included in Table S1.

## SUPPLEMENTAL MATERIAL

Supplemental material spectrum.02628-25-s0001.docx Supplemental figure legends.

Fig. S1 and S2 spectrum.02628-25-s0002.tiff CHS4 mRNA stability is unaffected by methyl-deficient mutation of Puf4-FLAG; PUF4 mRNA and protein stability are unaffected by methyl-deficient mutation of Puf4-FLAG.

Fig. S3 spectrum.02628-25-s0003.tiff LAC1 mRNA stability is not regulated by Puf4 or methyl-deficient mutants.

Fig. S4 and S5 spectrum.02628-25-s0004.tiff Pearson correlation of IP-MS replicates; Upset plots for protein interactions.

Table S1 spectrum.02628-25-s0005.xlsx Proteomic data and oligonucleotide sequences.

ASM does not own the copyrights to Supplemental Material that may be linked to, or accessed through, an article. The authors have granted ASM a non-exclusive, world-wide license to publish the Supplemental Material files. Please contact the corresponding author directly for reuse.
