# Supplementary material for: Puf4 is methylated and exhibits a temperature-dependent interactome in Cryptococcus neoformans
Source: Microbiol Spectr. 2026 Feb 25;14(4):e02628-25. doi: 10.1128/spectrum.02628-25 (PMC13055242; doi:10.1128/spectrum.02628-25)
Supplement: Supplemental material — Supplemental figure legends. [file spectrum.02628-25-s0001.docx]

**Supplemental Figure Legends.**

**Figure S1: *CHS4* mRNA stability is unaffected by methyl-deficient mutation of Puf4-FLAG**

**Figure S2: PUF4 mRNA and protein stability are** **unaffected by methyl-deficient mutation of Puf4-FLAG.** (a) Schematic depicting the location of a Puf4-binding element in the *PUF4* mRNA 5’ UTR. (b) *PUF4* mRNA stability is unchanged by 462 RtoK and 783/783 RtoK Puf4-FLAG mutations. (c) Puf4-FLAG protein stability is unchanged by 462 RtoK and 783/783 RtoK Puf4-FLAG mutations.

**Figure S3: *LAC1* mRNA stability is not regulated by Puf4 or methyl-deficient mutants.**

**Figure S4: Pearson correlation of IP-MS replicates.** Pearson correlation coefficient was calculated for targets that were included in the heatmap shown in Figure 7b.

**Figure S5: Upset plots for protein interactions identified in at least one of two replicates (R1**Ս**R2)**
