# Supplementary figures and images for: Puf4 is methylated and exhibits a temperature-dependent interactome in Cryptococcus neoformans
Source: Microbiol Spectr. 2026 Feb 25;14(4):e02628-25. doi: 10.1128/spectrum.02628-25 (PMC13055242; doi:10.1128/spectrum.02628-25)

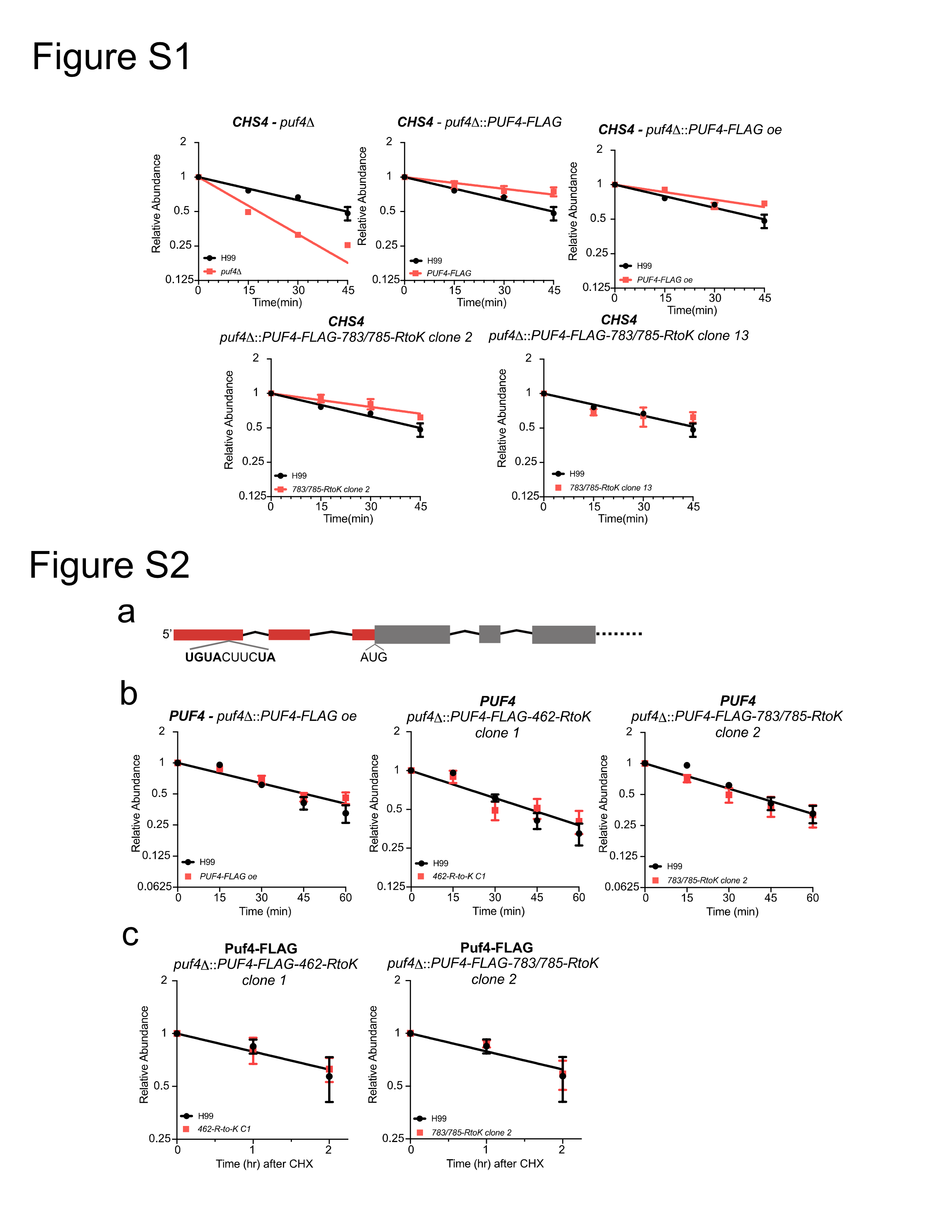

Supplement: Fig. S1 and S2 — CHS4 mRNA stability is unaffected by methyl-deficient mutation of Puf4-FLAG; PUF4 mRNA and protein stability are unaffected by methyl-deficient mutation of Puf4-FLAG. [file spectrum.02628-25-s0002.tiff]

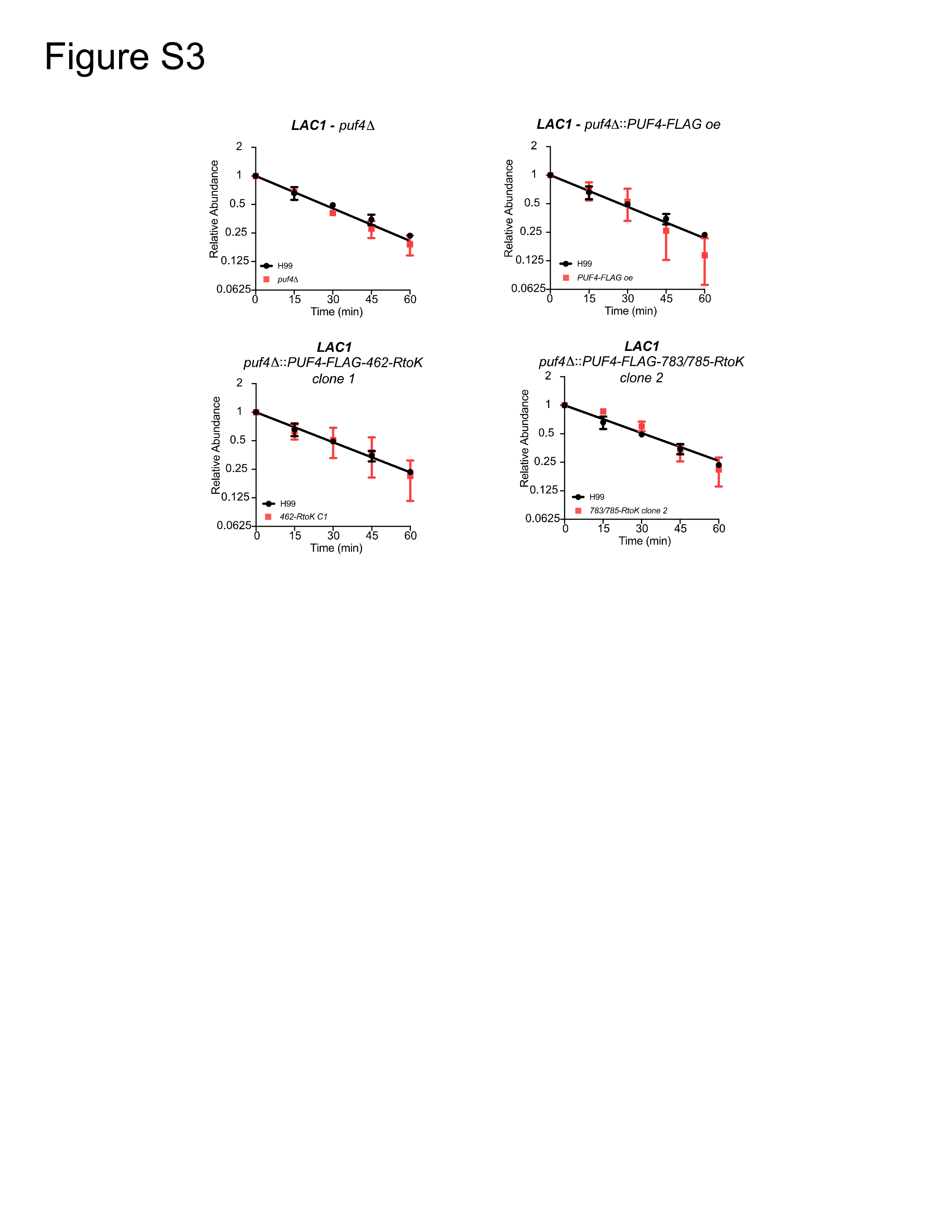

Supplement: Fig. S3 — LAC1 mRNA stability is not regulated by Puf4 or methyl-deficient mutants. [file spectrum.02628-25-s0003.tiff]

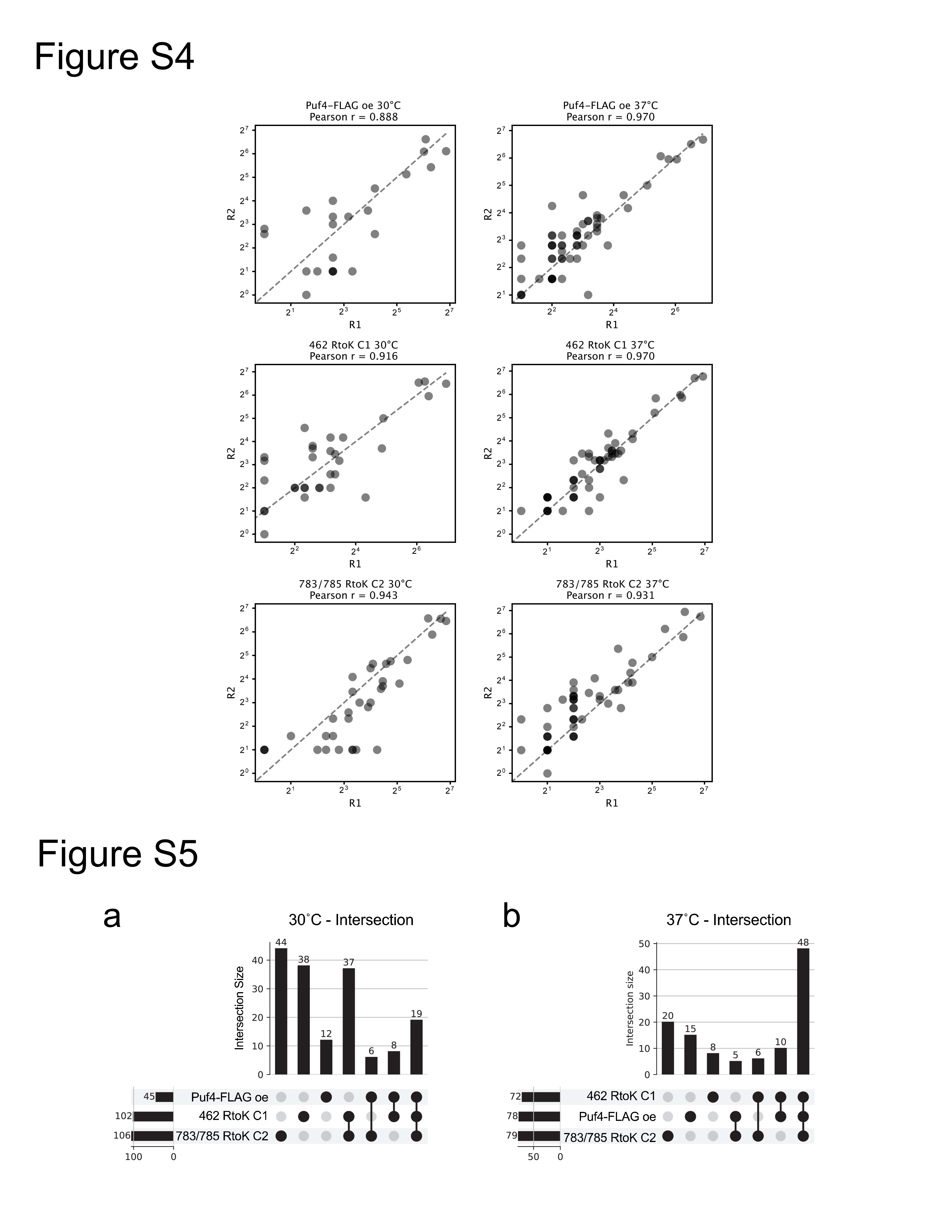

Supplement: Fig. S4 and S5 — Pearson correlation of IP-MS replicates; Upset plots for protein interactions. [file spectrum.02628-25-s0004.tiff]
